# Inter-rater reliability of kinesthetic measurements with the KINARM robotic exoskeleton

**DOI:** 10.1186/s12984-017-0260-z

**Published:** 2017-05-22

**Authors:** Jennifer A. Semrau, Troy M. Herter, Stephen H. Scott, Sean P. Dukelow

**Affiliations:** 1 Hotchkiss Brain Institute, University of Calgary, Calgary, AB Canada; 2 Department of Clinical Neurosciences, University of Calgary, Calgary, AB Canada; 3 Department of Exercise Science, University of South Carolina, Columbia, SC USA; 4 Department of Anatomy and Cell Biology, Queen’s University, Kingston, ON Canada; 5 Foothills Medical Centre, South Tower – Room 905, 1403 29th St NW, Calgary, AB T2N 2T9 Canada

**Keywords:** Proprioception, Kinesthesia, Stroke, Robotics, Sensorimotor, Inter-rater reliability

## Abstract

**Background:**

Kinesthesia (sense of limb movement) has been extremely difficult to measure objectively, especially in individuals who have survived a stroke. The development of valid and reliable measurements for proprioception is important to developing a better understanding of proprioceptive impairments after stroke and their impact on the ability to perform daily activities. We recently developed a robotic task to evaluate kinesthetic deficits after stroke and found that the majority (~60%) of stroke survivors exhibit significant deficits in kinesthesia within the first 10 days post-stroke. Here we aim to determine the inter-rater reliability of this robotic kinesthetic matching task.

**Methods:**

Twenty-five neurologically intact control subjects and 15 individuals with first-time stroke were evaluated on a robotic kinesthetic matching task (KIN). Subjects sat in a robotic exoskeleton with their arms supported against gravity. In the KIN task, the robot moved the subjects’ stroke-affected arm at a preset speed, direction and distance. As soon as subjects felt the robot begin to move their affected arm, they matched the robot movement with the unaffected arm. Subjects were tested in two sessions on the KIN task: initial session and then a second session (within an average of 18.2 ± 13.8 h of the initial session for stroke subjects), which were supervised by different technicians. The task was performed both with and without the use of vision in both sessions. We evaluated intra-class correlations of spatial and temporal parameters derived from the KIN task to determine the reliability of the robotic task.

**Results:**

We evaluated 8 spatial and temporal parameters that quantify kinesthetic behavior. We found that the parameters exhibited moderate to high intra-class correlations between the initial and retest conditions (Range, *r*-value = [0.53–0.97]).

**Conclusions:**

The robotic KIN task exhibited good inter-rater reliability. This validates the KIN task as a reliable, objective method for quantifying kinesthesia after stroke.

## Background

The identification and measurement of sensorimotor deficits after stroke has historically placed considerable focus on motor impairment. Proprioceptive deficits (our sense of limb position and motion [1]) have received far less attention in both research and clinical practice. Evidence has shown that sensory impairments occur in the majority of stroke survivors [2–5], and are thought to negatively impact functional ability and recovery after stroke [6–9]. Further, differences have been found in the timing and trajectory of motor and proprioceptive recoveries after stroke [5]. Our understanding of proprioceptive impairments after stroke has been limited by the fact that proprioception is difficult to measure using standard clinical examinations [10].

Clinical assessments for measuring impairments in proprioception typically detect only the most severe impairments. They often rely on the examiner to move a body segment (e.g., the finger) and ask the subject whether the finger has been moved upward or downward [11]. Other tests, such as the Thumb Localizer Test [12], rely on the examiner to position the thumb of the affected arm above the head and have the patient locate their thumb, without vision, using their unaffected arm. These clinical assessments often have poor sensitivity because they collapse across different components of proprioception (position sense and kinesthesia) and often utilize simplistic 2- or 3-point ordinal scales [10, 12].

Furthermore, these proprioceptive assessments have been shown to have low reliability among assessors [10]. Efforts to shorten clinical evaluation time of longer and more thorough sensory assessments, such as the Nottingham Sensory Assessment, has been shown to negatively impact inter-rater reliability [13]. Further, due to the limited numerical range of measurement, the Nottingham is susceptible to floor and ceiling effects similar to measures of motor impairment (Fugl-Meyer) [14] that exhibit reduced detection of and sensitivity to sensorimotor impairment.

New methodology and technology for assessment has taken steps to improve measurement of proprioceptive function after stroke [3, 4, 15, 16]. The use of robotics for assessing sensorimotor impairment has gained significant popularity [3, 4, 17–19] due to the ability to obtain objective, reliable, sensitive measurements capable of detecting sensorimotor deficits that clinical measures often miss [20]. In addition, robotic assessments can be completed relatively rapidly without the need for a clinician to be present. However, for these measures to be determined reliable, it is necessary to test the reproducibility of results. This can be influenced by intrinsic subject variability, as well as factors related to operator setup of the subject in the robot.

Developing measures that accurately and reliably assess proprioception after stroke is important, because both proprioceptive and motor deficits have been found to be significantly correlated with the performance of activities of daily living following stroke [8, 21]. To better inform neurorehabilitation therapies and practices, there is a need for more sensitive and reliable measurement tools for identifying proprioceptive impairments after stroke. Our previous study investigating kinesthetic deficits after stroke [4] did not examine the reliability of the kinesthetic robotic measure. Good reliability is a critical component to any clinical test to be sure that it can properly evaluate change over time. To examine reliably of the robotic kinesthesia task we quantified performance in neurologically normal subjects and subjects with stroke that performed the same task with multiple operators to evaluate the reliability of the task.

## Methods

We evaluated inter-rater reliability of a previously described kinesthetic matching task (KIN) [4, 5, 22]. We evaluated kinesthetic behavior in 25 neurologically-intact control subjects and 15 individuals with first-time stroke. To be included in the study, all subjects had to be aged 18 years or older. For subjects with stroke, inclusion criteria required them to have first-time, clinically identified unilateral stroke. Subjects with stroke were excluded if they had aphasia, apraxia or significant cognitive impairments that limited them from understanding three-step instructions. Neurologically-intact control subjects were recruited from the Calgary community. Stroke subjects were recruited from the acute stroke and rehabilitation units at Foothills Hospital in Calgary. The study was approved by the University of Calgary Ethics Board, and all subjects provided informed consent.

### Robotic kinesthesia task

**Fig. 1:**
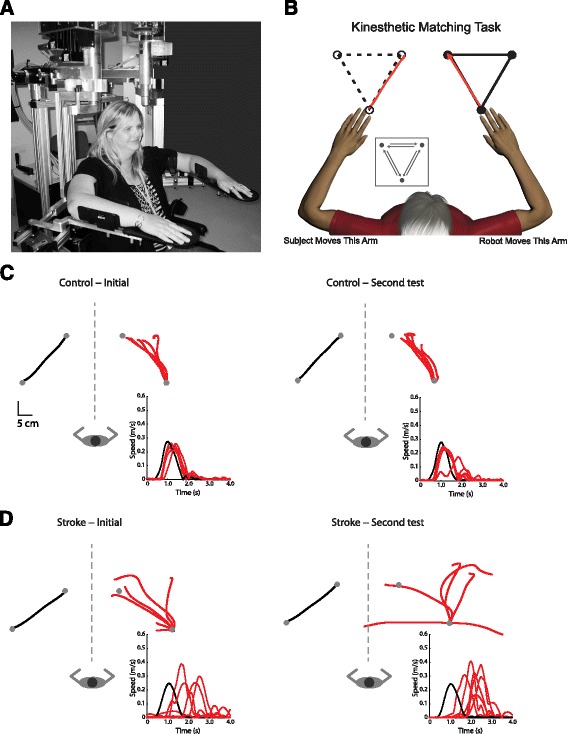
**a** Picture of the KINARM robotic exoskeleton. **b** Cartoon diagram of the kinesthetic matching (KIN) task. The robot moved the subjects’ stroke-affected arm and subjects matched the direction, speed and magnitude of movement with their opposite arm as soon as they felt the robot begin to move. Exemplar data from the KIN task for a neurologically intact subject (**c**) and a subject with stroke (**d**) for both initial and second test

Subjects were seated in the robotic exoskeleton (Fig. 1a) with their arms supported by gravity. Each subject was custom-fitted and calibrated in the robot based on their limb geometry by one of three experienced robot operators.

In brief, in the KIN task (Fig. 1b) the robot moved the subjects’ stroke-affected arm at a predetermined speed, direction and distance. For each trial, subjects were instructed to mirror-match the direction while matching the speed and magnitude of the robotic movement as soon as they felt the robot begin to move. Neurologically-intact subjects were tested on both the dominant and non-dominant arms.

Subjects were initially set up in the robot by one rater (initial time-point) and the subject completed the KIN robot assessment one time without the use of vision and one time with visual feedback of the limbs. The condition without vision always preceded the condition with vision to avoid the potential confound of subjects using visual cues about target location learned in the condition with vision.

At a second time-point, subjects were run in a second session, where the subject was custom fitted by a second robot operator. Subjects then completed the KIN task again, both with and without the use of vision. Three subjects with stroke did not complete the KIN task in the condition with vision (*N* = 12), due to subject time constraints.

### Robotic and statistical analyses

For each subject, we computed the mean of 8 robotic parameters across the 36 trials to quantify kinesthetic performance [4]: 1) Initial Direction Error (IDE) – angular deviation relative to the direction of the robotic movement; 2) Path Length Ratio (PLR) – length of matching movement relative to the length of the robotic movement; 3) Response Latency (RL) – time to initiate a matching movement in response to the robot movement, 4) Peak Speed Ratio (PSR) – peak speed of the matching movement relative to the peak speed of the robotic movement. We also calculated the variabilities for each of the individual parameters to evaluate consistency of error (IDEv, PLRv, RLv, PSRv). To evaluate the inter-rater reliability of each parameter, we computed two-way random average measures intra-class correlations (ICCs) [23].

To determine overall task performance, we computed normalized z-scores for each parameter. These scores were compared to 95% normative ranges derived from a large sample of neurologically intact subjects (*N* = 166), a group that includes the control subjects described in this study. We considered the potential influence of age, sex and handedness on task performance [5]. If a subject scored outside of the 95% range (one-tailed, z > 1.65), they were determined to have failed the individual parameter, as lying outside the 95% range indicates that behavior on that parameter was significantly different from controls. For overall task performance, subjects who failed more than 2 out of 8 parameters were determined to have failed the task. This failure threshold was determined based on the fact that only 5% of the sample of 166 neurologically intact subjects fall outside the normative range on 3 or more parameters.

### Clinical assessments

Subjects in both the neurologically intact and stroke groups were evaluated for handedness with the Edinburgh Handedness Inventory [24]. Subjects with stroke were evaluated on a variety of clinical measures: 1) Functional Independence Measure, which measures functional ability in motor and cognitive domains, and is scored out of 126 [25]; 2) Behavioural Inattention Test, which evaluates the presence or absence of visuospatial deficits via six conventional subtests (line bisection, letter cancellation, star cancellation, line cancellation, figure copying and drawing) and is scored out of 146 [26]; 3) Thumb Localization Test, which measures proprioceptive impairment, and is scored on a 4-point scale (0 indicates intact ability to find the thumb, 1 indicates ability to locate the thumb via locating the wrist, 2 indicates ability to locate the thumb via locating the arm, and 3 indicates completely unable to locate the thumb) [12]; 4) Chedoke-McMaster Stroke Assessment, which measures motor impairment of the arm and hand, and is scored on a 7-point scale (1 = flaccid paralysis, 2 = no voluntary movement, but spasticity present, 3 = marked spasticity and synergy patterns, 4 = decrease in spasticity and synergy patterns, 5 = mild spasticity, synergy pattern present but can be reversed, 6 = indicates near normal movement, 7 = indicates normal movement) [27]), 5) Purdue Pegboard, which evaluates manual dexterity by requiring subjects to insert as many pegs into holes as they can in 30 sec [28].

## Results

The neurologically-intact group (*N* = 25) was an average age of 38.3 ± 13.0 (SD) years old, 17 subjects were female, eight subjects were male, 22 were right-handed, and three were left-handed. The stroke group (*N* = 15) (Table 1) was an average age of 54.5 ± 13.6 (SD) years old, three subjects were female, 12 subjects were male, 14 subjects were right-handed, and 1 subject was left-handed. Subjects with stroke were tested, on average 62.4 ± 63.4 days post-stroke. One subject (subject 10) had visuospatial neglect as determined by the Behavioral Inattention Test. This subject was also classified as having moderate (≥40 or ≤ 80) impairment on the Functional Independence Measure. All other subjects scored within the mild functional impairment range (>80) on the Functional Independence Measure. The time from initial session to the retest session of the robot for neurologically-intact subjects was 1.3 h (median, range = [0.6–192.4 h]) and 21.5 h for stroke subjects (range = [0.3–52.8 h], Table 1). Clinical scores for stroke subjects are reported in Table 1.

**Table 1:** Clinical demographics for stroke subjects

Stroke subject	Affected side	Days post- stroke	Hours elapsed initial-retest	Stroke type	Lesion location	Vascular territory	FIM	BIT	TLT	CMSA [arm, hand]	PPB
1	R	4	19.0	I	SC	MCA	82	142	0	[4, 4]	0
2	R	7	22.6	I	C	MCA	101	138	0	[4, 5]	4
3	B	7	24.2	I	Cb	AICA	95	145	0	[6, 7]	5(R)^a^
4	B	10	0.3	I	Cb + Br	PICA	110	144	0	[7, 7]	10(L)^b^
5	R	14	6.6	I	C + SC	MCA	124	139	0	[7, 7]	12
6	R	18	0.8	I	C + SC	MCA	94	135	2	[5, 6]	4
7	L	21	24.9	I	C	MCA	74	141	3	[1, 1]	0
8	L	43	23.8	I	SC	MCA	106	135	1	[4, 6]	7
9	L	50	20.9	I	SC	MCA	115	142	0	[5, 6]	8
10	L	83	21.5	I	SC	MCA	85	128	1	[2, 2]	0
11	R	86	52.8	H	C + SC	MCA	111	144	1	[3, 4]	0
12	R	87	24.2	H	C + SC	MCA	106	146	0	[5, 6]	10
13	R	147	25.1	I	C	MCA	124	142	0	[7, 7]	11
14	L	178	5.1	H	SC	MCA	115	144	2	[3, 5]	0
15	R	186	0.8	I	C	MCA	126	145	0	[7, 7]	12

*Abbreviations*: *L* left, *R* right, *B* bilateral, *I* ischemic, *H* hemorrhagic, *C* cortical, *SC* subcortical, *Cb* cerebellar, *Br* brainstem, *MCA* middle cerebral artery, *AICA* anterior inferior cerebellar artery, *PICA* posterior inferior cerebellar artery, *FIM* Functional Independence Measure, *BIT* Behavioral Inattention Test, *TLT* thumb localizing test, *CMSA* Chedoke-McMaster Stroke Assessment, *PPB* Purdue Pegboard

^a^Subject 3 scored 6 for both arms and 7 for both hands on the CMSA, PPB is reported for the more impaired (right) arm

^b^Subject 4 scored 7 for both arms and hands on the CMSA, PPB is reported for the more impaired (left) arm

We compared overall robotic performance for the initial and retest sessions for neurologically-intact subjects and subjects with stroke. We found that, in the no vision condition, one neurologically-intact subject failed the KIN task (failed > 2 parameters) in the initial session, but no neurologically intact subjects failed the KIN task in the retest session. In comparison, we found that 40% (*N* = 6) of stroke subjects failed the KIN task in both the initial and retest sessions.

### Inter-rater reliability of robotic measures

Figure 1c presents the results of an exemplar neurologically-intact subject during the initial (left panel) and retest (right panel) sessions in the KIN task without vision. Performance on the KIN task was similar for this subject on the initial session (RL = 241.0 ms, PSR = 1.2, IDE = 13.7°, PLR = 1.1) and the second session (RL = 260.6 ms, PSR = 0.96, IDE = 13.5°, PLR = 1.0). In comparison, the subject with stroke (Fig. 1d) qualitatively shows obvious impairment in the initial and second session. The subject with stroke, however, also had similar results in both the initial session (RL = 1299.5 ms, PSR = 1.3, IDE = 32.1°, PLR = 1.3) and second session (RL = 1078.5 ms, PSR = 1.5, IDE = 35.3°, PLR = 1.5).

**Fig. 2:**
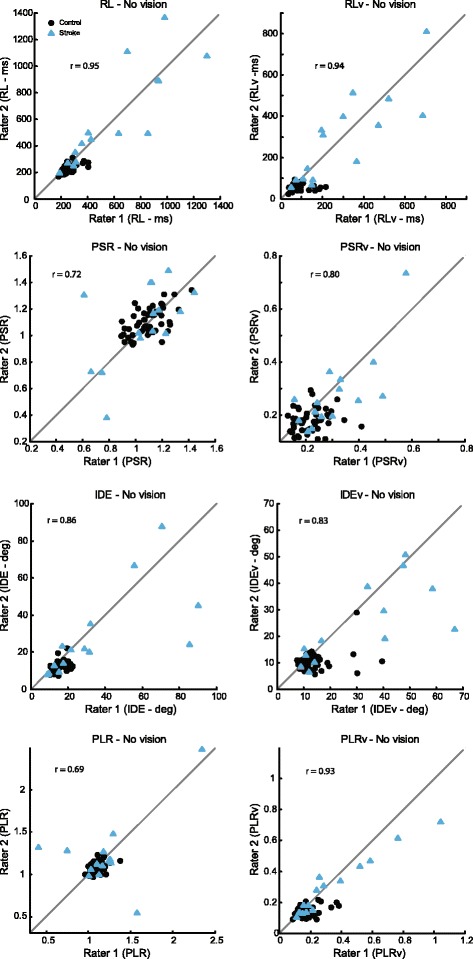
Intra-class correlations (ICCs) for the 8 parameters in the KIN task for performance without the use of vision. *R*-values for ICCs for the KIN no-vision condition were very high and ranged from 0.69 to 0.95

**Fig. 3:**
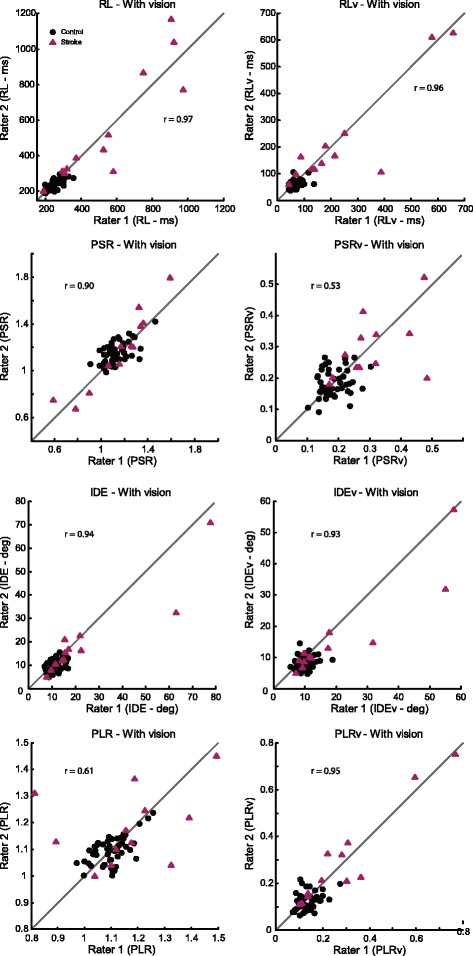
Intra-class correlations (ICCs) for the 8 parameters in the KIN task for performance when subjects were given full vision of their limbs. Similar to the no vision condition, test-retest reliability was generally high, with *r*-values ranging from 0.53 to 0.97

When we examined the relationship between initial and second performance for each of the 8 parameters in KIN without vision, we found that most parameters had high ICCs (Fig. 2, Table 2) (*r*-values, RL = 0.95, RLv = 0.94, PSR = 0.72, PSRv = 0.80, IDE = 0.86, IDEv = 0.83, PLR = 0.69, PLRv = 0.93). When subjects completed the task with the use of vision, we found that ICCs of most KIN parameters were similarly high (Fig. 3) (*r*-values, RL = 0.97, RLv = 0.96, PSR = 0.90, PSRv = 0.53, IDE = 0.94, IDEv = 0.93, PLR = 0.61, PLRv = 0.95).

**Table 2:** Calculated ICCs (*r*-values) for all subjects (Control + Stroke), neurologically-intact only subjects (Control), and subjects with stroke (Stroke)

Parameter	Group		
NO VISION	Control + Stroke	Control	Stroke
IDE	0.86^*^	0.52^*^	0.81^*^
IDEv	0.83^*^	0.41	0.84^*^
PLR	0.69^*^	0.59^*^	0.68
PLRv	0.93^*^	0.52^*^	0.95^*^
RL	0.95^*^	0.72^*^	0.92^*^
RLv	0.94^*^	0.10	0.91^*^
PSR	0.72^*^	0.72^*^	0.71
PSRv	0.80^*^	0.30	0.86^*^
VISION			
IDE	0.94^*^	0.64^*^	0.95^*^
IDEv	0.93^*^	0.37	0.94^*^
PLR	0.61^*^	0.73^*^	0.44
PLRv	0.95^*^	0.49	0.97^*^
RL	0.97^*^	0.83^*^	0.94^*^
RLv	0.96^*^	0.57	0.95^*^
PSR	0.90^*^	0.71^*^	0.96^*^
PSRv	0.77^*^	0.41	0.66

^*^indicates *p* < 0.0063, Bonferroni corrected (*n* = 8, α = 0.05)

We examined the inter-rater reliability of each of the subject groups independently and generally found that inter-rater reliability was higher for the stroke group compared to neurologically-intact controls in both the No Vision and Vision conditions (Table 2).

## Discussion

A major issue in the field of neurorehabilitation is that there is generally a poor understanding of the characteristics and recovery of proprioceptive deficits after stroke. These deficits are often difficult to detect and measure clinically, and can even be mistaken for motor deficits [29]. Currently, there is no gold standard for evaluating proprioception or its’ sub-modalities (position sense, kinesthesia) [1] after stroke. Further, it is thought that many of the current clinical measures of sensory impairment are not sensitive enough to detect clinically meaningful changes in proprioceptive function over time [30, 31], necessitating the development of new more sensitive measurement tools (i.e., robotics). Clinical tests, such as the Thumb Localizer Test, test position sense directly, and subcomponents of other clinical tests (Rivermead Assessment of Somatosensory Performance, Nottingham Sensory Assessment, Fugl-Meyer) evaluate elements of proprioception. Typically, the inter-rater reliability of many of these measures have been reported anywhere from poor to excellent [13, 32, 33]. Oftentimes, authors will limit an evaluation scale (0–2 vs 0–10), which generally leads to better reliability. However, this is clinically problematic because it typically produces a concomitant decrease in sensitivity to detect change.

Here we present a reliable robotic tool that can identify deficits in kinesthesia after stroke. Performance on the assessment has been previously shown to correlate with a number of clinical measures (e.g., Functional Independence Measure, Chedoke-McMaster Stroke Assessment, etc.) [4, 5, 22]. In the present manuscript, we focused on evaluating the inter-rater reliability of the robotic kinesthetic matching task. In general, we found that the inter-rater reliability for parameters within the KIN task was very high. We believe that developing objective and sensitive tools for measuring proprioception can significantly improve knowledge of proprioceptive impairment after stroke and can be applied to neurorehabilitation practice.

In testing inter-rater reliability, we are evaluating consistency of results when different individuals operate the robot. Over the years, many bedside clinical measures have been examined for both intra- and inter- rater reliability. The Nottingham Sensory Assessment, which evaluates several aspects of sensory function (tactile and kinesthetic sensation), has previously demonstrated poor inter-rater reliability [10]. Other investigators have specifically examined inter-rater reliability of the proprioceptive components of the Rivermead Assessment of Somatosensory Performance [32] and the Nottingham Sensory Assessment [13]. Their findings demonstrated low to fair agreement on many proprioceptive parameters with values ranging from *r* = 0.25 to 0.36 for the Rivermead (average = 0.31) and κ = 0.31–0.73 (average = 0.49) for the Nottingham. Other measures and methodologies for evaluating proprioception have shown fair to good test-retest reliability [34–36], but these measures also tend to rely on ordinal scales that are typically less sensitive to specific components of proprioceptive impairment. Our robotic parameters have, on average, very high inter-rater agreement and utilize continuous scales which should prove to be more sensitive than the simplistic ordinal scales used in the Rivermead or the Nottingham. Another advantage is the ability to evaluate specific kinematic aspects of kinesthesia (e.g., impairment in matching sensed movement length vs impairment in matching sensed movement speed).

Establishing a kinesthetic measure with high inter-rater reliability, as with the robotic kinesthesia task, is important for advancing the use of objective measures that are sensitive to impairments in proprioception. The development of such assessments is pivotal to advancing our understanding of post-stroke impairment and recovery. It is also vital for application of this knowledge to neurorehabilitative practices and therapies. Our robotic kinesthesia task allows for accurate and reliable measurement of kinesthesia following stroke. An advantage of this task is that it utilizes objective, continuous data that easily allows for comparison of individuals with stroke to neurologically-intact controls. However, a limitation of this task is that we observe that some parameters perform better than others when tested for inter-rater reliability (e.g., PLR (without vision), *r* = 0.69, Fig. 2; PSRv (with vision), *r* = 0.53, Fig. 3), it is possible that these parameters are more susceptible to within subject variability and may be less robust indicators of kinesthetic reliability. However, these values are still higher than those reported for most of the existing clinical measures of proprioception.

## Conclusions

We find that our robotic measurement of kinesthesia has very good inter-rater reliability in neurologically intact subjects and individuals with stroke. Validation of reliable, objective methods for quantifying kinesthesia in stroke is important in order to aide future identification of specific stroke-related impairments in proprioception. We believe this robotic assessment of kinesthetic impairment will aid in future identification of specific stroke-related impairments and will help us to better understand how various stroke-related impairments in kinesthesia contribute to functional deficits both on their own and in combination with motor impairments. This is significant because it allows us to identify whether potential treatments for kinesthesia are effective as well as allowing a better understanding of how various impairments in kinesthesia change over time in response to interventions.

## Abbreviations

ICC: Intra-class correlation
IDE: Initial direction error
IDEv: Initial direction error variability
KIN: Kinesthetic matching task
PLR: Path length ratio
PLRv: Path length ratio variability
PSR: Peak speed ratio
PSRv: Peak speed ratio variability
RL: Response latency
RLv: Response latency variability
TLT: Thumb localizer test
